# Detection and quantification of beta cells by PET imaging: why clinical implementation has never been closer

**DOI:** 10.1007/s00125-018-4745-5

**Published:** 2018-10-03

**Authors:** Martin Gotthardt, Decio L. Eizirik, Henk-Jan Aanstoot, Olle Korsgren, Dick Mul, Frank Martin, Marti Boss, Tom J. P. Jansen, Sanne A. M. van Lith, Mijke Buitinga, Olof Eriksson, Miriam Cnop, Maarten Brom

**Affiliations:** 1 Department of Radiology and Nuclear Medicine, Radboud university medical centre, PO Box 9101, 6500 HB Nijmegen, the Netherlands; 2 ULB Center for Diabetes Research, Medical Faculty, Université Libre de Bruxelles, Brussels, Belgium; 3 Diabeter, Center for Pediatric and Adolescent Diabetes Care and Research, Rotterdam, the Netherlands; 4 Rudbeck Laboratory, Department of Immunology, Genetics and Pathology, Uppsala University, Uppsala, Sweden; 5 JDRF, New York, NY USA; 6 Clinical and Experimental Endocrinology, University of Leuven, Leuven, Belgium; 7 Department of Medicinal Chemistry, Uppsala University, Uppsala, Sweden; 8 Division of Endocrinology, Erasmus Hospital, Université Libre de Bruxelles, Brussels, Belgium

**Keywords:** Clinical diabetes, Imaging, Islets, PET imaging

## Abstract

In this issue of *Diabetologia*, Alavi and Werner (10.1007/s00125-018-4676-1) criticise the attempts to use positron emission tomography (PET) for in vivo imaging of pancreatic beta cells, which they consider as ‘futile’. In support of this strong statement, they point out the limitations of PET imaging, which they believe render beta cell mass impossible to estimate using this method. In our view, the Alavi and Werner presentation of the technical limitations of PET imaging does not reflect the current state of the art, which leads them to questionable conclusions towards the feasibility of beta cell imaging using this approach. Here, we put forward arguments in favour of continuing the development of innovative technologies enabling in vivo imaging of pancreatic beta cells and concisely present the current state of the art regarding putative technical limitations of PET imaging. Indeed, far from being a ‘futile’ effort, we demonstrate that beta cell imaging is now closer than ever to becoming a long-awaited clinical reality.

## Introduction

In their commentary on the clinical study published by Cline and colleagues [1, 2], Alavi and Werner criticise the use of positron emission tomography (PET) for the imaging of pancreatic beta cells and put forward the argument that imaging of native beta cells is not technically feasible using this approach. They suggest that the research community should instead focus on efforts to image transplanted islets and beta cell mass in individuals with hyperinsulinism.

The arguments given by Alavi and Werner regarding the technical limitations of radiotracers and PET scanning [2] do not reflect the technical progress achieved in the field in the last decade. Furthermore, and as discussed below, these authors appear to have misinterpreted some of the publications referred to in their commentary. Here, we not only counter the statements regarding the technical limitations of PET, but also highlight the tremendous scientific potential of beta cell imaging using this method. The ongoing efforts by the scientific community that are aimed at in vivo visualisation of pancreatic beta cells are extremely relevant to the understanding of the natural history of type 1 and type 2 diabetes [3]. Beta cell dysfunction and loss of beta cell mass are the key pathophysiological factors in the development of type 1 and type 2 diabetes. Currently, beta cell mass can only be reliably measured by in vivo quantitative imaging based on PET scanning, and this could be combined with determination of beta cell function by clinical tests.

Our knowledge about diabetes has shifted towards a heterogeneous disease model involving a combination of both beta cell destruction and dysfunction. In this context, cellular stress factors within the beta cell are crucial aspects in the disease process (followed or preceded by autoimmunity in type 1 diabetes). Clinical studies using beta cell mass imaging, in combination with functional testing, would help us to determine the type of individuals and the phase of disease progression in which beta cell mass is lost and/or beta cell dysfunction plays a prominent role [3, 4].

Here, we put forward arguments to demonstrate that PET imaging is a powerful technology, which, at this point in time, offers an optimal combination of critical characteristics that enable the imaging of pancreatic beta cells. Furthermore, we refer to developments in the field of PET imaging that will further support the role of PET as a leading technology for the imaging of pancreatic beta cells, as well as islet grafts.

## Spatial resolution is not a primary issue for beta cell imaging—chemical resolution is the key to success

Alavi and Werner imply that beta cell imaging is a matter of identifying and then counting objects that are smaller than voxel size [2], which would indeed be impossible when faced with poor spatial resolution. This argument may be true in oncology, where identification of small objects such as, for example, peritoneal tumour deposits or small lymph node metastases, is the aim of imaging. In this case, spatial resolution is a limiting factor. The aim of beta cell imaging, however, is not to count beta cells/islets of Langerhans as dark spots on a light pancreas.

The optimal approach to beta cell imaging is to rely on detection of a signal that is specifically derived from the beta cells (‘chemical resolution’) with no or very low signal from the exocrine pancreas. PET is not only highly sensitive for the detection of low molar amounts of radiotracers (several orders of magnitude more sensitive than, for example, magnetic resonance imaging) [5], it also offers reliable quantification of global radiotracer uptake [6–8]. For the concept of chemical resolution, in addition to low background activity, very high uptake in the target tissue is of great importance. Metabolic trapping, i.e. trapping of a tracer molecule in the target tissues while it is washed out of non-target tissues, leads to improved image quality. For example, this occurs when using radiometal-labelled radiotracers, such as exendin derivatives [8]. When stating that ‘efforts to image targets that are dispersed within high background activity sites will fail’ [2], Alavi and Werner do not take into account the concept of chemical resolution based on beta cell-specific tracers with high uptake in beta cells and low uptake in the exocrine pancreas [8]. Alavi and Werner criticise the limited specificity of [^18^F]fluoropropyl-(+)-dihydrotetrabenazine (^18^F-FP-(+)-DTBZ) in this respect [2], but this critique should not be generalised to other tracers that are much more specific for the endocrine pancreas/beta cells (such as hydroxy-tryptophan (HTP) or exendin derivatives) [8].

In line with this, it is surprising that Alavi and Werner favour imaging of transplanted pancreatic islets using PET; these islets are usually dispersed in the liver, an organ often involved in radiotracer metabolism, which leads to high background activity and clearly represents a less favourable condition than the imaging of pancreatic beta cells located in their native site.

## How do we quantify beta cell mass by PET?

For beta cell imaging, the organ of interest (i.e. the pancreas) is large relative to voxel size. Any voxel in the pancreas may contain no to many islets. If there is high uptake of the tracer in the beta cells and no or low background signal (chemical resolution), the approach for quantification of a beta cell-specific signal is trivial because the signal correlates with specific binding and would yield a quantitative estimate of target density. If then the pancreas size is measured, the total beta cell mass can be calculated based on pancreatic uptake and pancreas size [8, 9]. The size of the pancreas, as determined by 3D imaging, ranges from 40 ml to 150 ml [10]. This represents considerable inter-individual variation, but it is obvious that in an organ of this size, and using current state-of-the-art PET scanners (with voxel sizes of usually 2–4 mm [6]), the partial volume effect will not have a relevant influence on the quantification of the total organ-derived signal.

Alavi and Werner state that breathing will lead to motion that prevents reliable quantification of pancreatic uptake [2]. This statement does not take into account that breath-gating technology has been introduced in PET imaging to correct for motion-derived artefacts and that current state-of-the-art breath gating significantly improves PET-based quantification [11]. PET imaging has been demonstrated to provide sufficient quantitative data to allow reliable diagnosis and individualised therapeutic interventions with excellent results [12].

## Literature has been misinterpreted

In their commentary [2], Alavi and Werner state ‘we hope that by continuing to communicate our views on this subject (which are shared by others)’, the scientific community will abandon attempts to image endogenous pancreatic islets by PET. They support this strong statement using a reference from some of the authors of the present commentary [8]. In the paper they refer to, we critically discuss the current status of radiotracer imaging of beta cells by PET and suggest how to proceed to validate existing tracer molecules. In no way did we suggest that beta cell imaging by PET is not feasible. On the contrary, and for the reasons described above, we strongly support the idea that PET is, at this point in time, the most promising technology for efficient in vivo imaging of beta cells. Although we critically discussed the limited specificity of dihydrotetrabenazine (DTBZ) tracers in this paper [8], we also clearly stated that other tracers provide better specificity. In addition, we do not believe that critical discussion of radiotracers targeting the tau protein [13] should be used to support the statement that current radiotracers are not specific or will not work for beta cell imaging. Tau protein imaging follows a completely different approach than imaging of specific internalising receptors, which allows both specificity and an extremely high target:background ratio through metabolic trapping, as mentioned above. In other words, a specific problem faced when using one radiotracer does not extend to the performance of other types of radiotracers, especially when the targeting principles are fundamentally different. Some excellent examples of specific radiotracers that enable the imaging of cells heterogeneously dispersed in a larger organ are the translocator protein (TSPO) ligands, which bind to activated microglia and astrocytes [14] for neuroinflammation imaging.

## Conclusion

In conclusion, in our opinion, Alavi and Werner’s discussion of PET imaging does not reflect the impressive technical developments in recent years, particularly following the introduction of integrated PET/computerised tomography (CT) systems. These improvements include widespread use of integrated PET/CT, as well as the introduction of PET/magnetic resonance (MR) (enabling precise motion correction for improved quantification), time-of-flight imaging and, most recently, digital PET systems [6, 7, 15, 16]. The current development of innovative technology to further improve the performance of clinical PET scanners, namely innovative detector technology and optimised system geometries, is expected to improve image quality in the near future, with spatial resolutions as low as 1–2 mm [6]. In addition, we expect that novel beta cell-specific ligands (for example, those binding to beta cell-specific proteins or highly specific protein splice variants) will provide us with a choice of novel radiotracers [17, 18]. In our view, and based on ongoing work by us and others, in vivo imaging of human pancreatic beta cells is close to become a clinical reality, which is also reflected by the number of ongoing clinical studies using PET for imaging of beta cells that can be found on www.clinicaltrials.gov (for example, NCT03182296, NCT02542059, NCT03182231). Rather than abandon this promising approach, we should redouble our efforts to reach the point, hopefully in the near future, where beta cell mass quantification may be included among the clinical tests used to evaluate and follow-up individuals with diabetes [3].

## Abbreviations

CT: Computerised tomography
PET: Positron emission tomography

## References

[1] Cline GW, Naganawa M, Chen L et al (2018) Decreased VMAT2 in the pancreas of humans with type 2 diabetes mellitus measured in vivo by PET imaging. Diabetologia. 10.1007/s00125-018-4624-0 10.1007/s00125-018-4624-0 29721633

[2] Alavi A, Werner TJ (2018) Futility of attempts to detect and quantify beta cells by PET imaging in the pancreas: why it is time to abandon the approach. Diabetologia. 10.1007/s00125-018-4676-1 10.1007/s00125-018-4676-1 29955934

[3] Gotthardt M, Eizirik DL, Cnop M, Brom M (2014). Beta cell imaging - a key tool in optimized diabetes prevention and treatment. Trends Endocrinol Metab.

[4] Dimeglio LA, Evans-Molina C, Oram RA (2018). Type 1 diabetes. Lancet.

[5] Catana C, Guimaraes AR, Rosen BR (2013). PET and MRI: the odd couple or a match made in heaven?. J Nucl Med.

[6] Berg E, Cherry SR (2018). Innovations in instrumentation for positron emission tomography. Semin Nucl Med.

[7] Jones T, Townsend D (2017). History and future technical innovation in positron emission tomography. J Med Imag.

[8] Eriksson O, Laughlin M, Brom M, Nuutila P, Roden M, Hwa A, Bonadonna R, Gotthardt M (2016). In vivo imaging of beta cells with radiotracers: state of the art, prospects and recommendations for development and use. Diabetologia.

[9] Brom Maarten, Woliner-van der Weg Wietske, Joosten Lieke, Frielink Cathelijne, Bouckenooghe Thomas, Rijken Paul, Andralojc Karolina, Göke Burkhard J., de Jong Marion, Eizirik Decio L., Béhé Martin, Lahoutte Tony, Oyen Wim J. G., Tack Cees J., Janssen Marcel, Boerman Otto C., Gotthardt Martin (2014). Non-invasive quantification of the beta cell mass by SPECT with 111In-labelled exendin. Diabetologia.

[10] Garcia TS, Rech TH, Bauerman Leitao C (2017). Pancreatic size and fat content in diabetes: a systematic review and meta-analysis of imaging studies. PLoS One.

[11] Grootjans W, de Geus-Oei LF, Meeuwis AP (2014). Amplitude-based optimal respiratory gating in positron emission tomography in patients with primary lung cancer. Eur Radiol.

[12] Gallamini A, Borra A (2014). Role of PET in lymphoma. Curr Treat Options in Oncol.

[13] Barrio JR (2018). The irony of PET tau probe specificity. J Nucl Med.

[14] Denora N, Natile G (2017). An updated view of translocator protein (TSPO). Int J Mol Sci.

[15] Bailey D. L., Pichler B. J., Gückel B., Barthel H., Beer A. J., Botnar R., Gillies R., Goh V., Gotthardt M., Hicks R. J., Lanzenberger R., la Fougere C., Lentschig M., Nekolla S. G., Niederdraenk T., Nikolaou K., Nuyts J., Olego D., Riklund K. Åhlström, Signore A., Schäfers M., Sossi V., Suminski M., Veit-Haibach P., Umutlu L., Wissmeyer M., Beyer T. (2016). Combined PET/MRI: from Status Quo to Status Go. Summary Report of the Fifth International Workshop on PET/MR Imaging; February 15–19, 2016; Tübingen, Germany. Molecular Imaging and Biology.

[16] Miller MA (2016) Focusing on high performance. https://philipsproductcontent.blob.core.windows.net/assets/20170523/360753349c5d4a6aa46ba77c015e75b4.pdf. Available from www.usa.philips.com/healthcare/product/HC882446/vereos-digital-petct-proven-accuracy-inspires-confidence. Accessed 12 August 2018

[17] Balhuizen A, Massa S, Mathijs I et al (2017) A nanobody-based tracer targeting DPP6 for non-invasive imaging of human pancreatic endocrine cells. Sci Rep 7(1):15130. 10.1038/s41598-017-15417-2 10.1038/s41598-017-15417-2 PMC5680294 29123178

[18] Eriksson Olof, Johnström Peter, Cselenyi Zsolt, Jahan Mahabuba, Selvaraju Ram K., Jensen-Waern Marianne, Takano Akihiro, Sörhede Winzell Maria, Halldin Christer, Skrtic Stanko, Korsgren Olle (2017). In Vivo Visualization of β-Cells by Targeting of GPR44. Diabetes.

